# Impact of ABO Blood Group on Bleeding and Clotting Time Among Healthy North Indian Males: A Pilot Study

**DOI:** 10.7759/cureus.99265

**Published:** 2025-12-15

**Authors:** Mritunjay Shukla, Shubham Patel, Nithya Janardhana, Waqas Alauddin

**Affiliations:** 1 Physiology, Naraina Medical College and Research Centre, Kanpur, IND; 2 Physiology, Chirayu Medical College and Hospital, Bhopal, IND; 3 Medicine, Adichunchanagiri Institute of Medical Sciences, Karnataka, IND

**Keywords:** abo blood-group system, clotting time, coagulation factor viii, haematology, haemostasis, von-willebrand factor

## Abstract

Background: The ABO blood group system plays a significant role in hemostasis through its influence on plasma von Willebrand factor (vWF) and factor VIII levels. Individuals with different ABO phenotypes exhibit variable coagulation profiles, potentially predisposing certain groups to bleeding or thrombotic tendencies. However, data focusing specifically on healthy North Indian males remains limited.

Objective: The objective of this study is to investigate the association between ABO blood types and hemostatic parameters, specifically clotting time (CT) and bleeding time (BT), in a group of healthy adult males from North India.

Methods: This cross-sectional study involved 200 healthy males aged 18 to 45 who were enrolled in the Department of Physiology of the Integral Institute of Medical Science and Research in Lucknow, India. The blood was grouped using the traditional slide agglutination technique. BT was measured using Duke's filter paper method, while CT was determined using the capillary tube method. Statistical analysis was conducted using IBM Corp. Released 2020. IBM SPSS Statistics for Windows, Version 26. Armonk, NY: IBM Corp. Continuous variables were summarized as mean ± SD. Group differences across ABO blood groups were evaluated using one-way ANOVA, followed by Tukey and Bonferroni post-hoc tests for significant results. A p-value < 0.05 was considered statistically significant.

Results: Among 200 healthy North Indian males analyzed, blood group B was the most common (36%), followed by O (29%), A (23%), and AB (12%), with a predominance of Rh-positive individuals (95.5%). Significant variation in hemostatic parameters was observed across ABO groups. Individuals with blood group O demonstrated the longest bleeding time (3.75 ± 0.49 min) and clotting time (6.45 ± 0.86 min), while groups A, B, and AB exhibited shorter values. One-way ANOVA confirmed significant intergroup differences for both bleeding time (p < 0.001) and clotting time (p < 0.001), whereas age, BMI, hemoglobin, and platelet count did not differ significantly across groups. Post-hoc analyses using Tukey and Bonferroni methods revealed that group O differed significantly from all non-O groups for both parameters. No significant differences were found among the A, B, and AB groups.

Conclusion: Blood group O individuals demonstrated significantly prolonged bleeding and clotting times compared with non-O groups, likely reflecting lower plasma levels of von Willebrand factor and factor VIII. These results highlight the influence of the ABO blood type on hemostatic balance and emphasize its potential clinical relevance in preoperative screening, transfusion management, and evaluation of bleeding risk.

## Introduction

The ABO blood group system, discovered by Karl Landsteiner in 1900, is the foundation of transfusion medicine and a crucial genetic factor for hemostatic function [1,2]. It also influences plasma proteins, including von Willebrand factor (vWF) and factor VIII, which maintain hemostatic balance [1,2]. The interaction between these factors and ABO antigens has gained increasing attention for its clinical and pathophysiological significance in the past decade [1,2].

Individuals of different ABO phenotypes exhibit marked variations in plasma vWF and factor VIII levels, with those of blood group O consistently demonstrating 25-30% lower concentrations than non-O counterparts [1,3]. This reduction translates into measurable alterations in bleeding and clotting times, potentially modulating the threshold between physiological hemostasis and pathological bleeding [4]. Such subtle yet consistent differences may influence individual responses to surgical trauma, anticoagulant therapy, and even cardiovascular risk, making ABO typing a potential indirect marker of coagulation efficiency [3,4].

ABO antigens affect the glycosylation pattern, secretion rate, and plasma half-life of vWF molecules [1]. In group O individuals, the absence of A or B carbohydrate moieties leads to accelerated vWF catabolism and reduced circulation stability, prolonging bleeding and clotting durations [5]. This observation highlights the subtle role of a classical genetic system in vascular homeostasis and coagulative resilience [4,5].

The North Indian population exhibits unique genetic diversity and ABO distribution patterns, with blood group B being the most prevalent phenotype [6]. However, regional data specifically addressing how ABO phenotypes influence hemostatic parameters such as bleeding time (BT) and clotting time (CT) in healthy adult males remain scarce. Given the known inter-ethnic variability in both vWF expression and coagulation physiology, it is plausible that population-specific trends exist within Indian cohorts that have yet to be characterized.

Understanding the relationship between ABO blood groups and basic hemostatic indices in this demographic may refine preoperative risk assessment, guide transfusion strategies, and contribute to the broader understanding of genetic determinants of coagulation dynamics. Hence, the present study was undertaken to evaluate the influence of ABO blood group on bleeding and clotting time among healthy North Indian males, providing novel insight into population-specific hemostatic variability and its potential clinical implications.

## Materials and methods

Between June and December 2021, this cross-sectional observational study was carried out in the Department of Physiology at Integral Institute of Medical Science and Research, Lucknow, North India. The Institutional Ethics Committee approved the study with ethical certificate number IEC/IIMS&R/2021/22. Before being included in the study, all participants provided written informed consent. The purpose of the study design was to evaluate the relationship between hemostatic parameters, specifically clotting time (CT) and bleeding time (BT), and ABO blood groups in a population of healthy adult males.

As this study was conducted as a pilot investigation, the sample size was determined on the basis of feasibility and the need to capture sufficient representation across the four ABO blood groups. Pilot studies are not expected to use formal power calculations; instead, they aim to estimate variability and assess methodological suitability. A sample of 200 healthy males was deemed adequate to generate reliable preliminary estimates of bleeding and clotting times and to support exploratory comparisons among ABO groups [7,8].

Through random sampling, 200 healthy male volunteers between the ages of 18 and 45 were chosen from among community volunteers, students, and hospital employees. Hormonal effects on coagulation dynamics, which are known to vary throughout menstrual and hormonal cycles, were reduced by the exclusive inclusion of males [9]. Individuals with a history of recent infections, hepatic or renal dysfunction, bleeding or coagulation disorders, anemia, or thrombocytopenia were not allowed to participate. To guarantee the validity of the results, people on drugs that interfere with platelet or coagulation function, such as anticoagulants, antiplatelet agents, nonsteroidal anti-inflammatory drugs (NSAIDs), or hormonal therapies, were also not included [10].

The standard slide agglutination technique, which has been validated and is commonly used in hematological studies, was used to perform blood grouping [11]. Aseptic peripheral venous blood samples from the fourth digit (digitus medicinalis) were obtained, and commercially available monoclonal anti-A, anti-B, and anti-D sera were used to determine both ABO and Rh typing. The corresponding antigenic reaction was indicated by the appearance of agglutination within two minutes. Where necessary, results were reverse grouped to ensure accuracy [11].

Bleeding time was measured using Duke's filter paper method, which is a standard bedside test for primary hemostasis [12]. After cleaning the fingertip with 70% isopropyl alcohol, a standard puncture was performed using a sterile lancet. The resulting blood was blotted onto filter paper at 30-second intervals without touching the wound. The number of minutes between the moment of puncture and the cessation of bleeding served as a proxy for BT [12]. 

Clotting time was measured using the capillary tube method [13]. About 0.5 milliliters of capillary blood was put into a glass capillary tube that had been sterilized and dried. The tube was gently broken every 30 seconds until a fine fibrin thread became apparent, which indicated that a clot had formed [13]. The number of minutes between collection and fibrin formation was recorded using CT [13]. To reduce inter-observer and environmental variation, both tests were conducted under standardized conditions in a temperature-controlled laboratory (25 ± 2°C).

Statistical analysis was performed using IBM Corp. Released 2020. IBM SPSS Statistics for Windows, Version 26. Armonk, NY: IBM Corp. Data were screened for completeness and accuracy prior to analysis. Continuous variables were expressed as mean ± standard deviation (SD). Blood groups were coded numerically (A = 1, B = 2, AB = 3, O = 4) for analytical purposes. Since assumptions for parametric analysis were met, group differences across the four ABO blood groups were analyzed using one-way analysis of variance (ANOVA). ANOVA results were reported with F statistics, degrees of freedom, and exact p-values. When a significant omnibus effect was observed, post-hoc pairwise comparisons were performed using both Tukey’s Honestly Significant Difference (HSD) test and Bonferroni correction to control multiple comparisons. Significance was set at p < 0.05 for all tests.

## Results

Descriptive statistics for key variables across ABO blood groups

A total of 200 healthy male participants were included in the analysis. Table 1 summarizes the descriptive statistics for demographic variables and hemostatic parameters across ABO blood groups. The mean age, BMI, hemoglobin concentration, and platelet count were comparable among the four groups, with no clinically meaningful variation. Baseline characteristics were therefore considered balanced across all groups. Bleeding time (BT) and clotting time (CT) demonstrated noticeable variation between ABO groups. Participants with blood group O exhibited a distinctly higher mean BT (3.75 ± 0.49 minutes) compared with groups A (2.95 ± 0.53 minutes), B (3.03 ± 0.62 minutes), and AB (2.80 ± 0.37 minutes). A similar pattern was observed for CT, with group O showing the longest mean CT (6.45 ± 0.86 minutes), followed by groups B (5.86 ± 0.63 minutes), A (5.76 ± 0.75 minutes), and AB (5.62 ± 0.42 minutes). 

**Table 1:** Descriptive statistics for key variables across ABO blood groups Table 1 presents the descriptive statistics for demographic variables and hemostatic parameters across the four ABO blood groups. Mean values for age, BMI, hemoglobin, and platelet count were comparable among groups, indicating balanced baseline characteristics. Bleeding time and clotting time showed notable variation, with group O demonstrating the highest mean values for both parameters. Data are expressed as mean ± standard deviation. n = numbers

Variable	Group A (n = 46) Mean ± SD	Group B (n = 72) Mean ± SD	Group AB (n = 24) Mean ± SD	Group O (n = 58) Mean ± SD	Total (n = 200) Mean ± SD
Age (years)	31.54 ± 8.84	30.94 ± 8.63	32.46 ± 8.87	31.18 ± 8.79	31.33 ± 8.70
BMI (kg/m²)	23.71 ± 3.16	24.22 ± 3.51	23.84 ± 3.29	23.56 ± 2.95	23.87 ± 3.24
Hemoglobin (g/dL)	14.92 ± 1.35	15.12 ± 1.39	14.78 ± 1.37	14.84 ± 1.12	14.95 ± 1.30
Platelet Count (×10³/µL)	275.22 ± 54.85	269.36 ± 52.80	259.28 ± 67.36	273.32 ± 42.65	270.63 ± 52.43
Bleeding Time (min)	2.95 ± 0.53	3.03 ± 0.62	2.80 ± 0.37	3.75 ± 0.49	3.19 ± 0.65
Clotting Time (min)	5.76 ± 0.75	5.86 ± 0.63	5.62 ± 0.42	6.45 ± 0.86	5.97 ± 0.77

**Figure 1:**
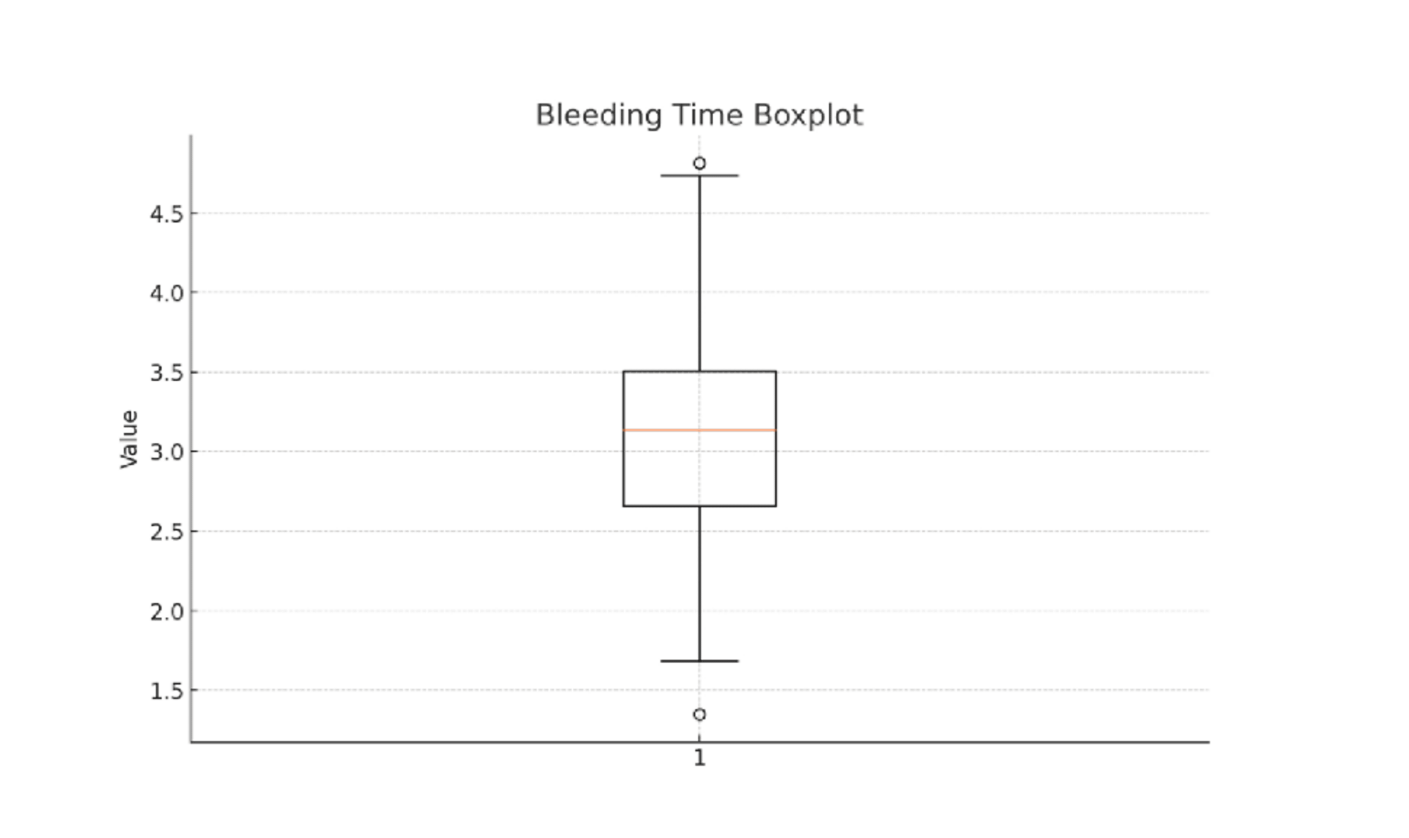
Boxplot representing the distribution of bleeding time (BT) among 200 healthy male participants The central box displays the interquartile range (IQR), spanning from the 25th percentile (Q1) to the 75th percentile (Q3). The horizontal line inside the box represents the median BT, while the whiskers extend to the minimum and maximum values within 1.5 × IQR. Values beyond the whiskers are plotted as outliers. In this sample, the median BT is approximately 3.1 minutes, with most values ranging from 2.7 to 3.5 minutes, indicating a relatively narrow distribution. A few outliers below 1.5 minutes and above 4.5 minutes indicate rare deviations, possibly due to individual variability in vascular or platelet function.

A boxplot of bleeding time demonstrated a relatively symmetrical distribution, with a median value of approximately 3.1 minutes and an interquartile range spanning 2.7-3.5 minutes (Figure 1). Most observations fell within this range, with a small number of low and high outliers noted. These findings support the normal distribution assumption used for parametric statistical testing. 

One-way ANOVA across ABO blood groups

The one-way ANOVA findings (Table 2) confirmed that age, BMI, hemoglobin, and platelet count did not differ significantly across ABO categories (p > 0.05 for all). In contrast, both BT (F = 29.18, p < 0.001) and CT (F = 12.50, p < 0.001) differed significantly among the four blood groups.

**Table 2:** One-way ANOVA across ABO blood groups Table 2 presents the one-way ANOVA results comparing demographic variables and hemostatic parameters across the four ABO blood groups. No significant differences were observed for age, BMI, hemoglobin, or platelet count. In contrast, bleeding time and clotting time differed significantly among groups, with p-values < 0.001, indicating that ABO blood group has a measurable influence on hemostatic parameters.

Variable**	F Statistic	df (between, within)	p-value	Interpretation
Age	0.195	3,195	0.900	Not significant
BMI	0.490	3,195	0.690	Not significant
Hemoglobin	0.698	3,195	0.555	Not significant
Platelet Count	0.553	3,195	0.646	Not significant
Bleeding Time	29.18	3,195	<0.001*	Significant
Clotting Time	12.50	3,195	<0.001*	Significant

Tukey and Bonferroni post-hoc comparisons of bleeding time

Post-hoc pairwise comparisons using both Tukey and Bonferroni corrections demonstrated that the significant variation in bleeding time observed across ABO groups was driven predominantly by differences between group O and all non-O groups. As shown in Table 3, group O exhibited markedly higher bleeding time compared with groups A, B, and AB (mean differences ranging from -0.72 to -0.95 minutes; p < 0.001 for all comparisons). In contrast, no significant differences were observed among the non-O groups themselves (A vs. B, A vs. AB, and B vs. AB; p > 0.05). These results indicate that prolongation of bleeding time is a characteristic feature uniquely associated with blood group O, whereas groups A, B, and AB display comparable hemostatic profiles.

**Table 3:** Tukey and Bonferroni post-hoc comparisons of bleeding time Table 3 presents the results of post-hoc pairwise comparisons for bleeding time across the four ABO blood groups using Tukey and Bonferroni correction methods. Significant differences were observed exclusively between group O and all non-O groups, with group O showing consistently higher bleeding time values (p < 0.001). No significant differences were detected among groups A, B, and AB, indicating that the prolongation of bleeding time is specific to the O phenotype.

Comparison	Mean Difference	Tukey p-value	Bonferroni p-value
A vs. B	-0.08	0.874	1.000
A vs. AB	+0.15	0.694	1.000
A vs. O	-0.80	<0.001*	<0.001*
B vs. AB	+0.23	0.290	0.467
B vs. O	-0.72	<0.001*	<0.001*
AB vs. O	-0.95	<0.001*	<0.001*

Tukey and Bonferroni post-hoc comparisons of clotting time

Post-hoc analyses for clotting time revealed a pattern consistent with that observed for bleeding time. As shown in Table 4, participants with blood group O had significantly longer clotting times compared with groups A, B, and AB (mean differences ranging from -0.59 to -0.83 minutes; p < 0.001 for all comparisons using both Tukey and Bonferroni corrections). No significant differences were detected among the non-O groups (A vs. B, A vs. AB, and B vs. AB; p > 0.05). These findings indicate that the prolongation of clotting time is specific to individuals with the O phenotype, while all non-O groups demonstrate comparable coagulation profiles.

**Table 4:** Tukey and Bonferroni post-hoc comparisons of clotting time Table 4 summarizes the post-hoc analysis of clotting time differences between ABO blood groups using Tukey and Bonferroni adjustments. Similar to bleeding time, clotting time was significantly prolonged in group O compared with groups A, B, and AB (p < 0.001), while no significant differences were observed among non-O groups. These findings confirm that group O exhibits a distinct hemostatic profile characterized by longer clotting duration.

Comparison	Mean Difference	Tukey p-value	Bonferroni p-value
A vs. B	-0.10	0.873	1.000
A vs. AB	+0.14	0.863	1.000
A vs. O	-0.69	<0.001*	<0.001*
B vs. AB	+0.24	0.475	0.906
B vs. O	-0.59	<0.001*	<0.001*
AB vs. O	-0.83	<0.001*	<0.001*

**Figure 2:**
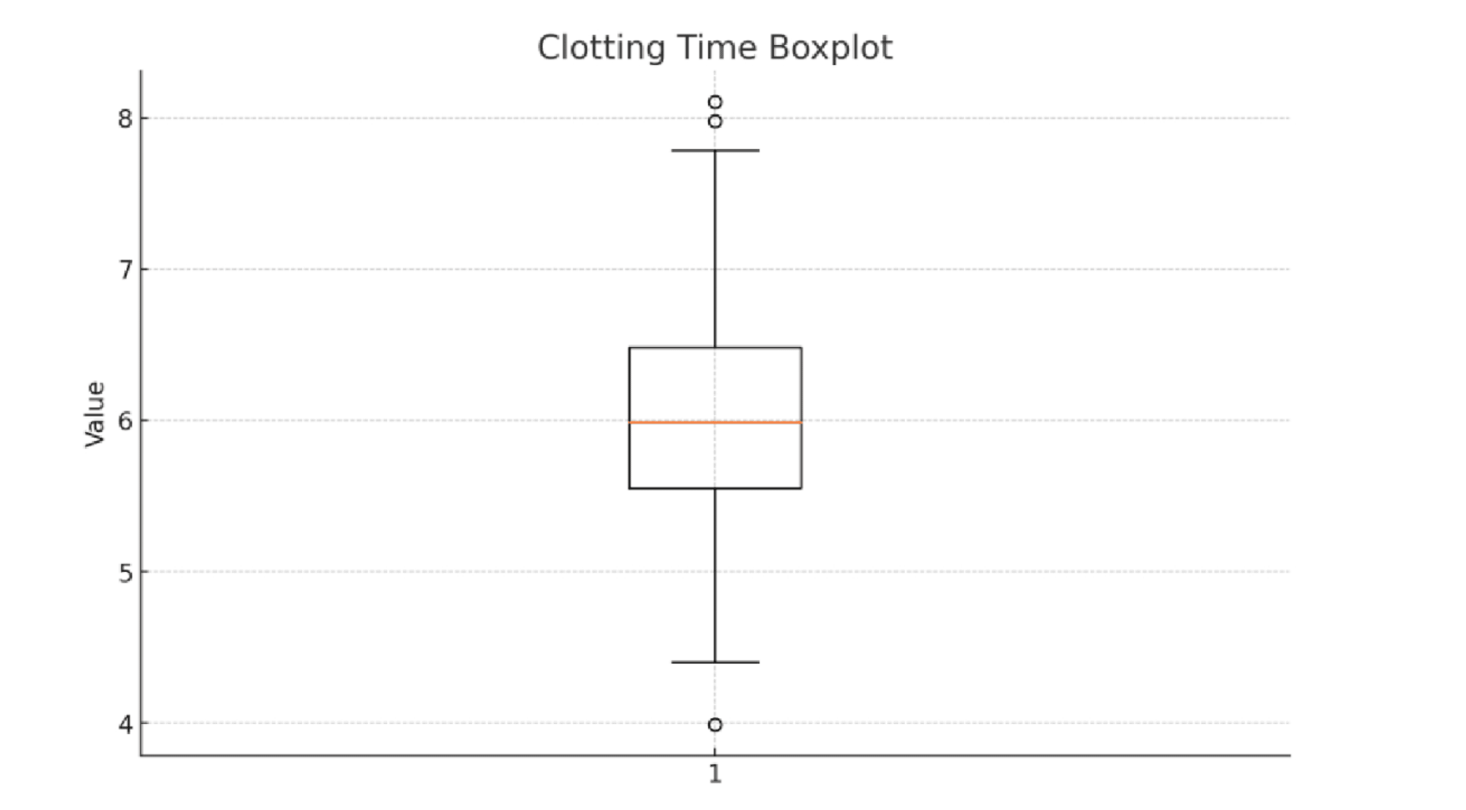
Boxplot representing the distribution of clotting time (CT) among 200 healthy male participants The box represents the interquartile range (IQR), extending from the 25th percentile (Q1) to the 75th percentile (Q3). The horizontal line within the box denotes the median CT value. The whiskers represent values within 1.5 × IQR, while individual points beyond the whiskers indicate outliers. In this cohort, the median CT is approximately 6.0 minutes, with the majority of values falling between 5.6 and 6.5 minutes. A small number of low (<4.5 minutes) and high (>7.5 minutes) outliers reflect individual variability in coagulation response.

A boxplot illustrating clotting time revealed a median CT of approximately 6.0 minutes, with an interquartile range of 5.6 to 6.5 minutes (Figure 2). The distribution was moderately symmetrical, with whiskers extending from approximately 4.4 to 7.7 minutes. A few outliers were noted below 4.0 minutes and above 8.0 minutes, indicating occasional deviations from typical coagulation patterns. Overall, the distribution supported the normality assumption for subsequent parametric testing.

## Discussion

Among healthy North Indian males, the current study showed a statistically significant correlation between hemostatic parameters and ABO blood groups. In line with earlier international and Indian findings, participants with blood group O showed noticeably longer bleeding and clotting times than non-O groups [6,14-17].

The primary physiological mechanism for this association is the impact of ABO antigens on von Willebrand factor (vWF) and factor VIII plasma levels [1]. This is because group O individuals lack A and B carbohydrate residues; vWF is cleared from circulation more quickly, which lowers its plasma concentration and lengthens bleeding and clotting times [1,3,14]. On the other hand, people in groups A and AB typically have higher levels of factor VIII and vWF, which facilitates quicker and more effective coagulation [3,4]. These results are consistent with those of Mehic et al. [14] and Chen et al. [18], who noted that group O participants had prolonged coagulation parameters. In our study, group O had increased bleeding time; this can be due to glycosylation patterns on GPIb in blood donors with blood group O. vWF demonstrated a lower binding rate for GPIb/vWF, indicating an ABO-dependent effect on platelet interaction, according to Dunne et al. [19].

However, in the current study, there was no statistically significant relationship between the Rh factor and either bleeding or clotting times. This is consistent with earlier findings by Mehic et al. [14] and Chen et al. [18] that Rh antigen expression has little effect on vWF synthesis or metabolism. The lack of detectable hemostatic effect can be explained by the fact that the Rh antigen system mainly influences the characteristics of red blood cell membranes instead of plasma protein dynamics.

From a clinical standpoint, understanding blood group-related variations in hemostasis is important for transfusion medicine, surgical risk assessment, and perioperative bleeding tendency prediction [13,14]. While people with non-O blood groups, particularly A and AB, may have a comparatively higher thrombotic potential, those with blood group O may be more likely to experience prolonged bleeding during invasive procedures [13,14]. Consequently, understanding this physiological variation can help direct tailored patient care and enhance clinical results.

The main limitation of this study is its restriction to male participants and reliance on conventional bedside techniques for assessing bleeding and clotting times. While these methods remain simple and cost-effective, they may not fully reflect quantitative plasma variations in vWF and factor VIII. In this study, no plasma vWF and factor VIII assays were performed. Future studies should include both genders, larger sample sizes, and advanced laboratory assays for vWF antigen, vWF activity, and factor VIII quantification to further elucidate the mechanistic pathways underlying these associations.

## Conclusions

This study confirms that males with blood group O exhibit significantly prolonged bleeding and clotting times compared to non-O groups, most likely due to genetically mediated reductions in plasma vWF and factor VIII activity. These findings underscore the role of the ABO system as a physiological determinant of hemostatic variability. Recognizing such differences is essential in clinical contexts, particularly during preoperative evaluations, transfusion planning, and the assessment of unexplained bleeding or bruising tendencies. Incorporating ABO-based hemostatic profiling into routine clinical assessment may improve risk stratification and personalized care.
